# Altered Prion Protein Expression Pattern in CSF as a Biomarker for Creutzfeldt-Jakob Disease

**DOI:** 10.1371/journal.pone.0036159

**Published:** 2012-04-27

**Authors:** Mauricio Torres, Luis Cartier, José Manuel Matamala, Nancy Hernández, Ute Woehlbier, Claudio Hetz

**Affiliations:** 1 Neuroscience Biomedical Institute, Faculty of Medicine, University of Chile, Santiago, Chile; 2 Center for Molecular Studies of the Cell, Institute of Biomedical Sciences, University of Chile, Santiago, Chile; 3 Department of Neurological Sciences, Faculty of Medicine, University of Chile, Santiago, Chile; 4 Neurounion Biomedical Foundation, Santiago, Chile; 5 Harvard School of Public Health, Boston, Massachusetts, United States of America; Nagasaki University Graduate School of Biomedical Sciences, Japan

## Abstract

Creutzfeldt-Jakob disease (CJD) is the most frequent human Prion-related disorder (PrD). The detection of 14-3-3 protein in the cerebrospinal fluid (CSF) is used as a molecular diagnostic criterion for patients clinically compatible with CJD. However, there is a pressing need for the identification of new reliable disease biomarkers. The pathological mechanisms leading to accumulation of 14-3-3 protein in CSF are not fully understood, however neuronal loss followed by cell lysis is assumed to cause the increase in 14-3-3 levels, which also occurs in conditions such as brain ischemia. Here we investigated the relation between the levels of 14-3-3 protein, Lactate dehydrogenase (LDH) activity and expression of the prion protein (PrP) in CSF of sporadic and familial CJD cases. Unexpectedly, we found normal levels of LDH activity in CJD cases with moderate levels of 14-3-3 protein. Increased LDH activity was only observed in a percentage of the CSF samples that also exhibited high 14-3-3 levels. Analysis of the PrP expression pattern in CSF revealed a reduction in PrP levels in all CJD cases, as well as marked changes in its glycosylation pattern. PrP present in CSF of CJD cases was sensitive to proteases. The alterations in PrP expression observed in CJD cases were not detected in other pathologies affecting the nervous system, including cases of dementia and tropical spastic paraparesis/HTLV-1 associated myelopathy (HAM/TSP). Time course analysis in several CJD patients revealed that 14-3-3 levels in CSF are dynamic and show a high degree of variability during the end stage of the disease. Post-mortem analysis of brain tissue also indicated that 14-3-3 protein is upregulated in neuronal cells, suggesting that its expression is modulated during the course of the disease. These results suggest that a combined analysis of 14-3-3 and PrP expression pattern in CSF is a reliable biomarker to confirm the clinical diagnosis of CJD patients and follow disease progression.

## Introduction

Protein misfolding is a common hallmark of several neurodegenerative disorders including Alzheimer's disease, Parkinson's disease, amyotrophic lateral sclerosis (ALS), as well as Prion-related disorders (PrDs), among other pathologies [1]. Although in many diseases, the molecular events underlying neurodegeneration has been elucidated, there is an urgent need for the identification of reliable biomarkers for early diagnosis and to follow disease progression in clinical trials. PrDs are the classic example of a protein misfolding disorder where alterations in the structure of the prion protein (PrP) lead to progressive degeneration [2], [3]. The most common PrD in humans is Creutzfeldt-Jakob disease (CJD), where the sporadic form (sCJD) accounts for nearly 85% of all cases with an incidence of 1 case per million individuals [4]. Familial CJDs (fCJD) are caused by mutations in the prion gene (*PRNP*) and represent in average 10% of all cases, whereas less than 1% of CJDs are infectious, highlighting new variant CJD, which is most likely associated with the consumption of cattle affected with bovine spongiform encephalopathy [5], [6]. In a few countries, including Chile, the incidence of CJD is considerably higher (>2.5 increase), where around 30% of all cases are familial associated with the E200K mutation of *PRNP* [7], [8]. PrDs lead to progressive fatal neurodegeneration, ultimately resulting in the patient's death within a few months of diagnosis [9]. Clinical manifestations of CJD include rapid progression of dementia, myoclonus, visual and cerebellar symptoms, pyramidial or extrapyramidial signs and akinetic mutism [10]. A definitive diagnosis of CJD can only be made after neuropathological examination of brain tissue and demonstration of spongiform degeneration of the brain, accompanied by extensive neuronal loss and accumulation of PrP^RES^, a misfolded and protease-resistant form of the normal cellular prion protein (PrP^C^) [11], [12]. Biochemical analysis of cerebrospinal fluid (CSF) is commonly used for clinical diagnosis of CJD. Increased levels of 14-3-3, tau protein, S100B or neuron-specific enolase are used to guide clinical diagnosis and to distinguish PrDs from other causes of rapidly progressive dementia. The importance of 14-3-3 protein for diagnosis has been widely discussed in the context of CJD [13]–[16]. However, contradictory findings regarding 14-3-3 test specificity and sensitivity have been reported (see examples in [17]–[19]). In fact an elevation of 14-3-3 levels in the CSF is thought to occur because of non-specific release from areas after massive brain damage, which accompanies a variety of neurological conditions including central nervous system infections and inflammatory diseases, brain tumors, and other types of neurodegenerative diseases [20]. These studies reflect the need for additional biomarkers for diagnosis and to follow disease progression in CJD, which represents a general need for most neurodegenerative diseases. Direct monitoring of PrP^RES^ in blood and CSF samples of CJD cases may represent a better indicator for diagnosis as previously reported based on the fact that alterations in this protein are the main cause of the disease [21]–[23]. Soluble PrP^C^ is present in high levels in CSF of healthy people, but lower concentrations of PrP^C^ are found in CSF of CJD patients [24], [25]. However, this was also observed in patients suffering from other unrelated neurodegenerative diseases [25].

In this study, we report the analysis of PrP^C^ levels in CSF in a large group of CJD patients. We found dramatic changes in the overall quantities and glycosylation pattern of PrP, which directly correlated with disease progression. Our data suggest that monitoring the pattern and levels of PrP^C^ in combination with measurements of 14-3-3 level in CSF of CJD patients may be used as a predictive biomarker for CJD diagnosis and progression.

## Results

### 14-3-3 protein and LDH levels in CSF of CJD patients

**Figure 1:**
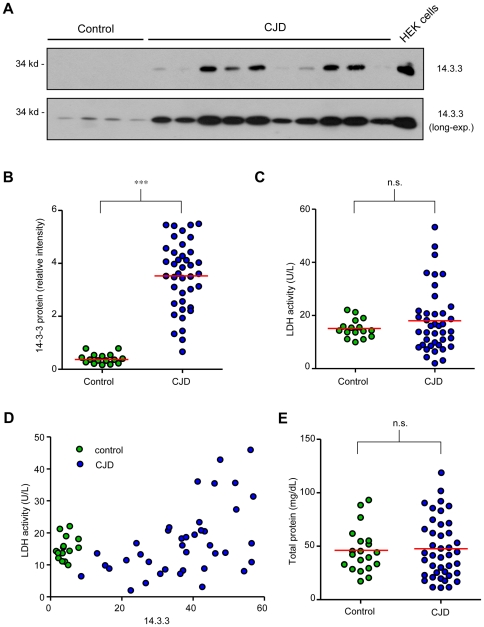
Low correlation between 14-3-3 protein levels and LDH activity in CSF of CJD patients. CSF samples from CJD patients (N = 40) and non-CJD controls (N = 16), were obtained by lumbar puncture. Then, **A.** 14-3-3 levels in CSF were analyzed by Western blot. **B.** Relative levels of 14-3-3 protein band were quantified using densitometric analysis. **C.** LDH activity was measured in each sample as described in Material and Methods. **D.** A correlation between 14-3-3 protein and LDH activity from panels B and C is presented. **E.** Quantitative analysis of total protein levels in CSF samples of panel A was performed. In B, C and E the red line represents the mean of all samples analyzed. Statistical significance was calculated using Student's t-test.

14-3-3 levels were monitored in CSF samples from CJD patients and control subjects by Western blot analysis (Figure 1A), followed by semi-quantitative analysis using densitometry (Figure 1B). Increased 14-3-3 levels were found in most CSF samples analyzed from CJD patients when compared with control subjects. We measured LDH activity in CSF as a marker for tissue disruption and found a moderate average increase in mean LDH activity in CJD patients compared to the control group (Figure 1C). However, LDH activity displayed broad variability between different CJD patient samples, whereas this parameter was highly homogeneous in the control group (Figure 1C). We found that increased LDH activity correlated only in patients with high 14-3-3 protein levels in CSF (Figure 1D). Examination of the total protein amount in CSF samples did not show significant differences between control subjects and CJD patients (Figure 1E).

### Altered PrP expression pattern in CSF of CJD cases

**Figure 2:**
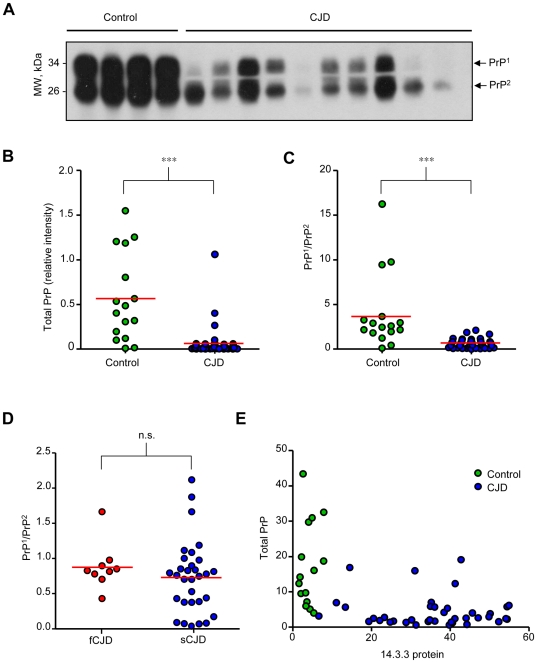
Altered PrP expression pattern in CSF of CJD patients. CSF samples from CJD patients (N = 40) and non-CJD controls (N = 16), were obtained by lumbar puncture. **A.** PrP levels in CSF were examined by Western blot. **B.** and **C.** Quantification of relative levels of total PrP protein in CSF and the ratio of the PrP band 1 (upper) and PrP band 2 (lower) in CSF, respectively. **D.** Comparison between ratios of PrP band 1 and 2 in sCJD versus fCJD **E.** Correlation of total PrP protein levels with 14-3-3 protein in each CSF samples presented in Fig 1. In B, C and D the red line represents the mean of all samples analyzed. Statistical significance was calculated using Student's t-test.

We then monitored the levels of PrP in CSF from CJD cases using Western blot analysis (Figures 2A and B). In control subjects, two main PrP bands with similar expression levels were detected possibly reflecting different glycosylation states (see below). A dramatic decrease in total PrP^C^ levels was observed in CJD-derived samples (Figure 2A). Unexpectedly, the ratio between the two main PrP forms present in CSF was drastically altered in most CJD patients, where the upper band was reduced, and in some patients was not even detectable (Figures 2A and C). We also compared the ratio between the two PrP bands in fCJD and sCJD cases, and did not observe significant differences between both groups of patients (Figure 2D). Finally, an inverse correlation was observed between 14-3-3 protein and total PrP levels in CSF of CJD patients compared to control subjects (Figure 2E). Similarly, an inverse correlation was observed between 14-3-3 levels and the PrP band ratio (data not shown).

**Figure 3:**
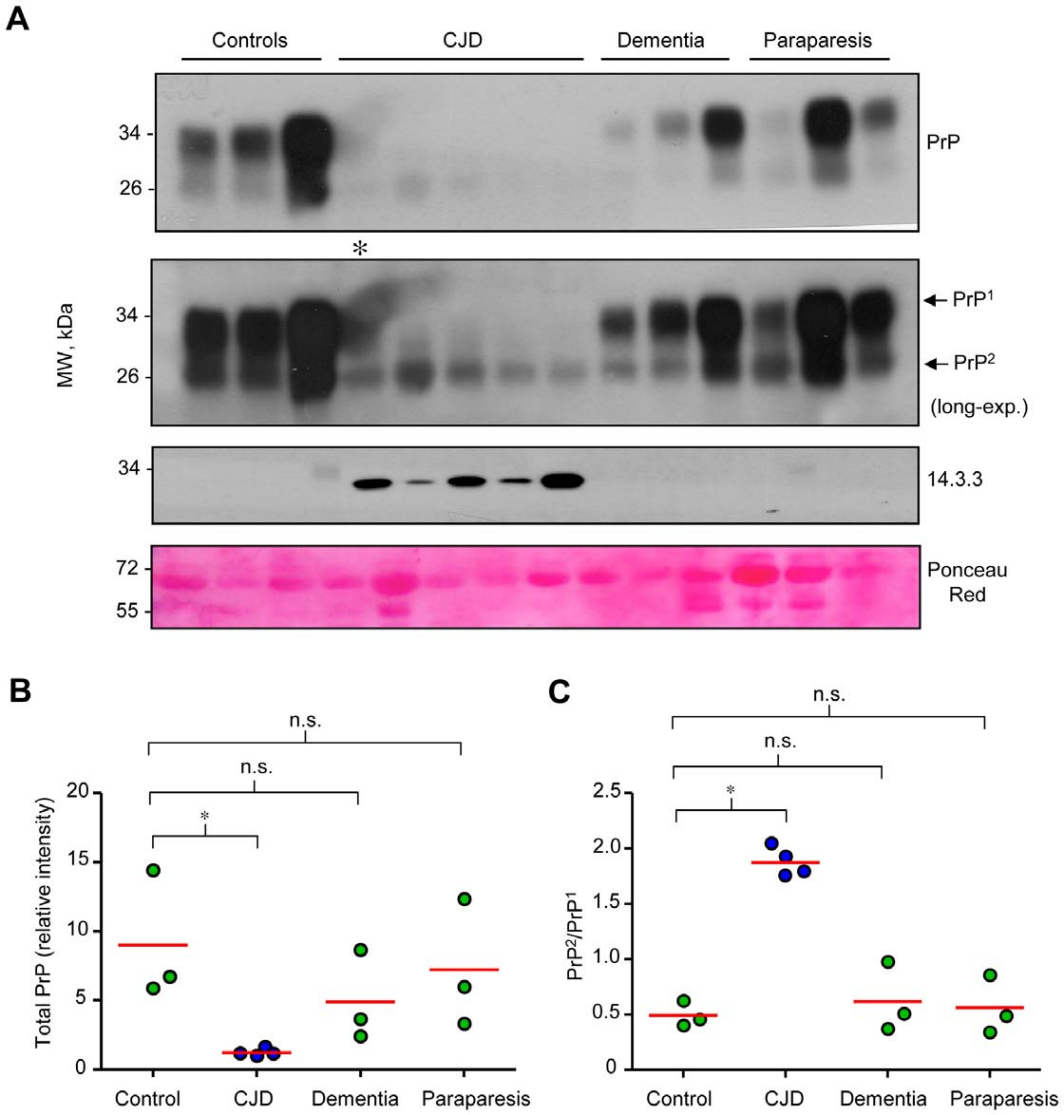
Alterations in PrP expression pattern in CSF are specific to CJD patients. CSF samples from healthy control subjects (N = 3), CJD patients (N = 5), dementia patients (N = 3) and paraparesis patients (N = 3), were obtained by lumbar puncture. **A.** Assessment of PrP levels (two upper panels with different exposure), and 14-3-3 protein levels (third panel) by Western blot analysis, and total protein content by Ponceau Red staining (lower panel, a selected band is presented). **B.** and **C.** Quantification of relative total PrP protein levels in CSF and the ratio of the PrP band 1 (upper) and PrP band 2 (lower) in CSF, respectively. The asterisk in A indicates one sample with a technical problem in the blot detection that was excluded for quantification of panel B and C. In B and C the red line represents the mean of all samples analyzed. Statistical significance was calculated using Student's t-test.

We performed additional controls to determine whether changes in 14-3-3 and PrP levels are specific for CJD pathology or can be observed in other conditions affecting the nervous system. We compared these parameters in CSF samples obtained from three healthy control individuals, five CJD patients, three non-CJD dementia patients (see exclusion criteria in Material and Methods) and three cases of HAM/TSP (Figure 3). A slight decrease in total PrP levels was observed only in two dementia patients and one HAM/TSP patient, but no increase in 14-3-3 protein was found (Figures 3A and B). The changes observed in the PrP band ratio detected in the analysis were not observed in this small set of non-CJD disease controls (Figure 3C).

### Altered PrP glycosylation pattern in CSF of CJD cases

**Figure 4:**
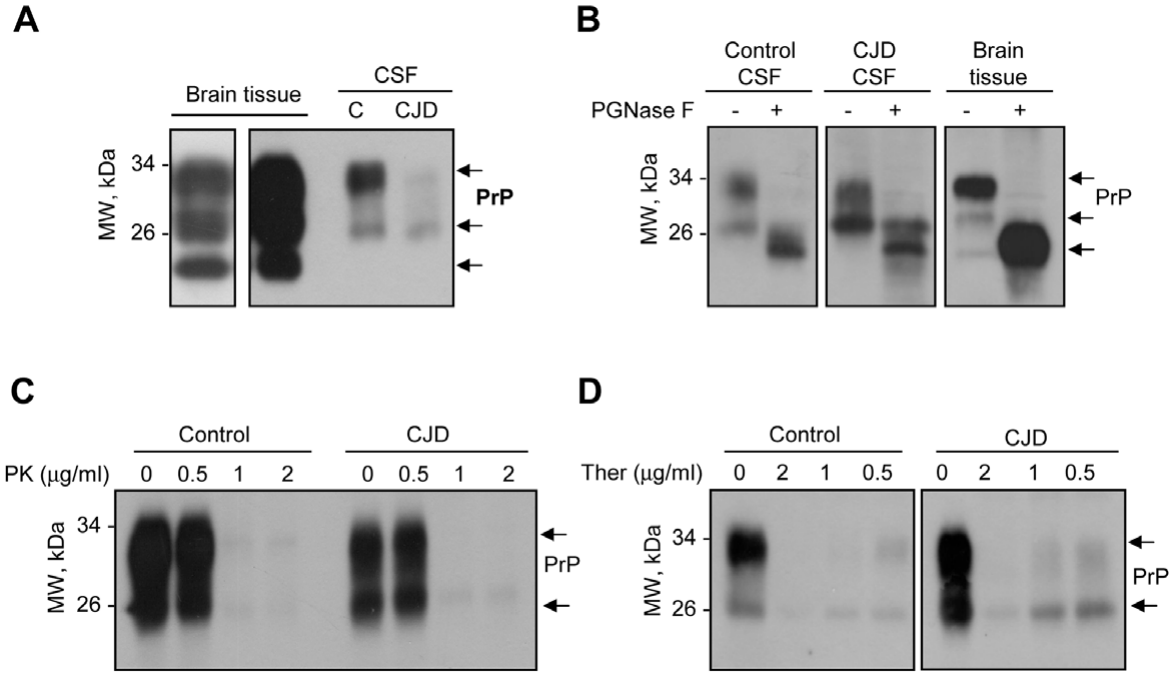
Changes in the glycosylation pattern of PrP in CSF of CJD patients. **A.** The pattern of expression of PrP in two representative CSF samples (from one healthy control individual and one CJD patient) is compared to the PrP pattern observed in control brain tissue using Western blot analysis. **B.** CSF samples from one healthy control individual, one CJD patient and one healthy control brain tissue were treated with PNGase F and then analyzed by Western blot. Data represent the analysis of three control and five individual CJD samples. **C.** and **D.** CSF samples from one healthy control individual and one CJD patient were treated with various concentrations of PK or thermolysin (Ther), respectively, followed by Western blot analysis. Data represent the analysis of three control and five individual CJD samples.

To address the possible cause of the changes in the ratio of the two PrP forms observed in CSF of CJD patients, we first compared the distribution of PrP bands in CSF with the pattern of human brain cortex tissue from healthy controls, which is known to contain the classical three PrP forms (mono-, di-, and non-glycosylated). Interestingly, this comparison revealed that the two bands present in CSF have a similar electrophoretic migration to the mono- and di-glycosylation PrP forms (Figure 4A). Samples were then treated with PNGase F (an unspecific glycosidase), which revealed the presence of mostly glycosylated PrP forms in CSF samples (Figure 4B).

A key biochemical signature of PrDs is the generation of PrP^RES^ in brain. We treated CSF samples from control and CJD patients with different concentrations of proteinase K (PK). With this method no PrP^RES^ species were detected even with very low levels of PK (Figure 4C). Additionally, we analyzed the sensitivity to thermolysin, a protease that can discriminate between normal and intermediate misfolded forms of PrP sensitive to PK [26]. Although we found a slight resistance of PrP to the treatment with this protease in CJD cases (Figure 4D), the vast majority of samples analyzed were sensitive to thermolysin (data not shown).

### Dynamic changes of PrP and 14-3-3 protein levels during the progression of CJD

**Figure 5:**
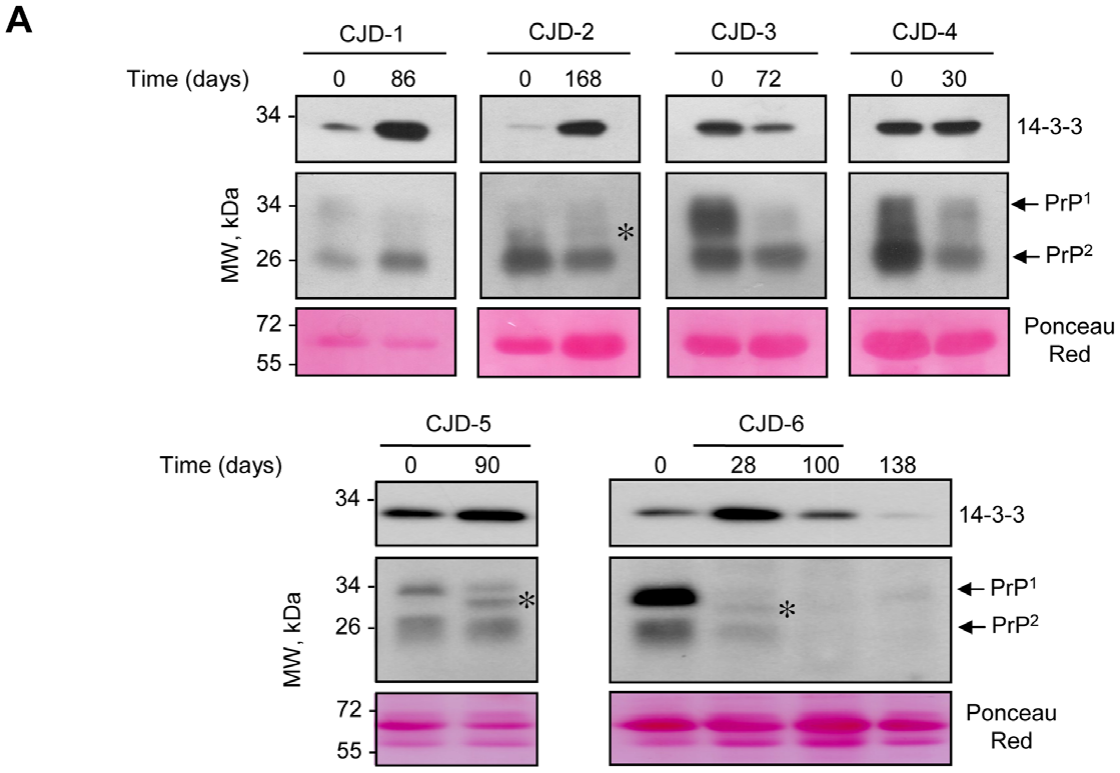
Changes in 14-3-3 protein and PrP levels in CSF from six CJD patients overtime. PrP and 14-3-3 protein levels were analyzed in CSF samples obtained by lumbar puncture of 6 CJD patients (CJD-1 to CJD-6) at different time points during disease. Total protein content was assessed by Ponceau Red staining, the predominant band (albumin) is shown. The asterisk in samples from CJD-2, CJD-5 and CJD-6 indicates an additional PrP protein band detected between PrP band 1 and band 2.

PrP and 14-3-3 protein levels were monitored in CSF from six CJD patients (CJD1-CJD6) over time at different disease stages (Figure 5). The first sample analyzed (point 0) corresponded to the time of clinical diagnosis of CJD. Unexpectedly, the overall 14-3-3 protein and PrP levels depicted clear differences when the same patient was studied over time, displaying distinct kinetics of changes with the course of the disease. In the CSF of patient 6, 14-3-3 levels peaked, then declined (Figure 5). In other CJD patients, an elevation of 14-3-3 protein levels was observed over time (patients 1, 2 and 5), whereas other patients showed increased levels at time of diagnosis and a reduction in 14-3-3 levels over time (patient 3) or were not altered over time (patient 4). Thus, the pattern of 14-3-3 expression is highly variable over time when the CSF of the same patient is analyzed at different time points of disease progression.

Overall the analysis of the PrP expression pattern over time showed robust and sustained changes (Figure 5). In contrast to 14-3-3 levels, changes in PrP expression levels and altered glycosylation pattern correlated with disease progression/severity (Figure 5). Of note, in some samples an additional band between PrP band 1 and band 2 was detected (CJD-2, CJD-5 and CJD-6), which may represent further changes in the PrP structure and/or post-translational modifications. Thus, these results indicate that alterations of total PrP levels and its glycosylation pattern have a predictive value for analysis of disease diagnosis and progression.

### Upregulation of 14-3-3 protein in post-mortem brain samples of CJD patients

**Figure 6:**
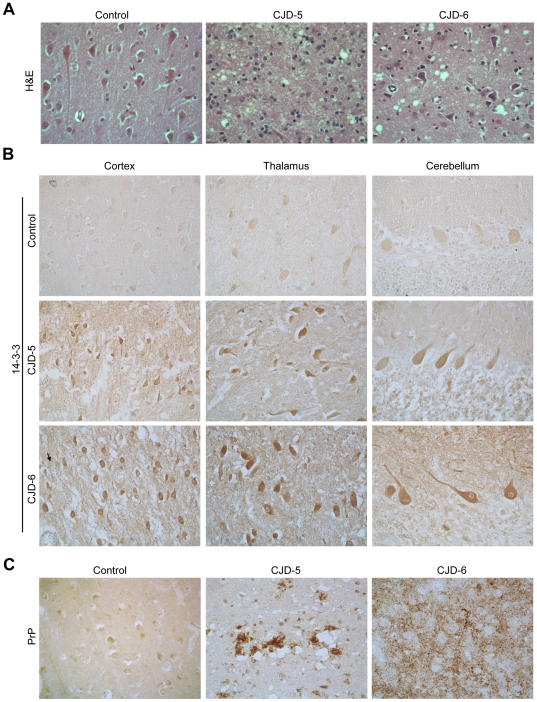
Postmortem examination of 14-3-3 in the brain of sCJD cases. **A.** H&E staining was performed in the post mortem brain cortex obtained from of CJD patients 5 and 6 (CJD-5 and CJD-6) and one patient with cerebral infarction (control, non-affected hemisphere). **B.** 14-3-3 protein expression levels were monitored by immunohistochemistry in post-mortem brain sections of patient CJD-5 and CJD-6 compared to a control subject. Brain cortex, thalamus and cerebellum were analyzed. **C.** In addition, the expression pattern of PrP was also analyzed in cortex presented in A. Magnification 400×.

To confirm the CJD diagnosis, we performed autopsies on two patients (CJD-5 and CJD-6) with complete histological analysis of the brain. The areas usually affected in CJD (cortex, thalamus and cerebellum) are shown in Figure 6. Patient CJD-6 died during the terminal stage of the disease (akinetic mutism), whereas patient CJD-5 suffered from an intrahospitalary infection, and died before entering in the terminal stage. Since the levels of 14-3-3 protein were altered in CSF over time, and LDH activity and total protein levels were in general not changed, we analyzed the expression pattern of 14-3-3 protein in brain tissue. The occurrence of spongiform degeneration in cerebral tissue confirmed the clinical diagnosis of CJD in these patients (Figure 6A). Remarkably, we found elevated levels of 14-3-3 protein in brain tissue of CJD patients compared with control subjects (Figure 6B). Higher expression of 14-3-3 protein was detected primarily in neurons and glia in the different brain areas (Figure 6B). In this brain region, an accumulation of PrP was also observed with large cores of extracellular PrP plaques surrounding areas of extensive spongiform change in patient CJD-5, whereas patient CJD-6 presented a more diffuse staining pattern (Fig. 6C). Despite differential prion patterns observed in the brain tissue of patients, we found similarly elevated levels of 14-3-3 protein.

## Discussion

This work was performed as part of a coordinated effort to develop a centralized diagnosis and surveillance of Chilean patients suffering from a prevalent human PrD form. Here, we present a full study that examines potential CSF biomarkers for CJD that can be used in combination with 14-3-3 protein. We monitored the levels of 14-3-3 protein, LDH activity and different aspects of PrP biology in the CSF of patients with the clinical diagnosis of CJD. The general consensus in the field indicates that although CSF 14-3-3 levels are not specific, in concert with characteristic clinical features, it may be used for the diagnosis of CJD. Here we report that total PrP levels combined with changes in its glycosylation pattern mirrors the disease progression in CJD. This is a significant finding since altered physicochemical properties of PrP underlie all PrDs. It is possible that the PrP expression pattern in CSF reflects the molecular events affecting PrP structure in the brain. Of note, an inverse relationship between the amount of 14-3-3 protein and total levels of PrP in the CSF was observed. When PrP levels were monitored in the same CJD patient over time, we observed a progressive decline in PrP levels paralleled by changes in the glycosylation pattern. As previously observed, PrP in CSF of CJD patients and non-CJD patients is sensitive to PK digestion [27]; however, with more sensitive methods, it is possible to detect minor amounts of abnormal PrP^RES^ conformations in CSF samples of the CJD cases [21], [23].

The biological function of soluble PrP^C^ and the cell types responsible for maintaining its levels in CSF are unknown. Some studies have shown that the soluble form of PrP participates in signal transduction, cell survival and protection against oxidative stress [28]–[30], suggesting a neuroprotective function of PrP in CSF. Therefore the loss of PrP in CSF may contribute to disease progression. Some studies argue that 14-3-3 levels are not necessarily related to the degree of histopathologic changes. It is recognized that the extent of spongiform degeneration can vary extensively among different CJD subtypes. Thus, 14-3-3 protein levels may only be helpful to diagnose classic CJD [31]. Non-classic sporadic CJD may be diagnostically challenging because of significantly longer disease duration and often heterogeneous symptoms. Since slower progression may reduce the sensitivity of the 14-3-3 test, some patients may require continued evaluation of 14-3-3 levels. In early studies, a high predictive value of the 14-3-3 test was reported [14]; nevertheless, it was later noted that the accuracy is reduced as the certainty of the clinical diagnosis decreases [32]. The 14-3-3 test has proven to be reliable when interpreted in the proper clinical context, since 14-3-3 protein positivity has been reported in a wide spectrum of vascular, inflammatory and neurodegenerative diseases [17], [33]. In addition, our variable results observed when 14-3-3 levels were monitored over time in CSF of the same CJD patients may help explain discrepancies previously published.

Despite initial predictions, we found normal levels of LDH activity and total protein concentration in CSF from confirmed CJD cases. Interestingly, post-mortem analysis revealed that, although there was a marked reduction in neurons in affected areas in CJD patients, 14-3-3 protein expression was significantly upregulated in brain tissue. Taken together, our results support the new concept that increased CSF 14-3-3 levels may reflect the induction of a cellular response against prion rather than cell lysis. In agreement with this hypothesis, it has been reported that some 14-3-3 protein isoforms may play a role in synaptic function [34]–[36]. In addition, 14-3-3 proteins function as conformational stabilizers of other proteins and regulate diverse signal transduction pathways related to cell survival [37]. 14-3-3 is also involved in axonal transport of prion proteins and their transport to the cell surface [38].

Changes in the glycosylation pattern of PrP in CSF may reflect alterations in the protein maturation pathways during its synthesis. PrP is generated and folded in the endoplasmic reticulum (ER) where it is subjected to several post-translational modifications, and then is transported to the Golgi apparatus to undergo maturation by glycosylations to be further exported to the plasma membrane [39]. Perturbations in the function of the ER have been described in human post-mortem samples of sCJD and new variant CJD [40], in addition to animal and cellular models of scrapie [41], [42]. Altered glycosylation of PrP also has been reported in the brains of many animal models of PrDs (see example in [43]), and all three PrP forms can reach the plasma membrane [44]. Based on these observations, we speculate that the changes in the PrP glycosylation pattern in CSF may reflect an active stress response at the level of the ER and secretory pathway of affected neurons in CJD. PrP levels may also decrease in CSF because it may cluster in large and stable aggregates in the brain due to PrP misfolding. Additionally, the results of Meyne et al., indicate that reduced PrP amounts in the CSF are observed in a wide range of neurodegenerative diseases [25]. In agreement with these results, a slight decrease in total PrP levels was observed in two dementia patients and one HAM/TSP patient, however, the changes in the ratio of PrP isoforms was observed only in the CJD patients analyzed.

To our knowledge, this study is the first report with comparative measurements of 14-3-3 protein, PrP levels and glycosylation pattern in CSF of CJD patients following these changes at different stages of disease progression. Our results suggest that analysis of 14-3-3 protein level and PrP expression pattern in CSF samples may be a reliable biomarker for diagnosis of CJD.

## Materials and Methods

### Patient samples and clinical diagnosis

CSF samples were collected from 46 patients diagnosed with CJD (sCJD, N = 35 patients and fCJD, N = 11 patients) and 16 anesthesia controls of healthy individuals (Table 1). Samples were collected, centrifuged and immediately frozen at −20°C for transport from the hospital to the laboratory for biochemical analysis. Then samples were stored as aliquots at −80°C. The clinical diagnosis of CJD was defined by the following parameters including: motor alterations, cerebellar signs, myoclonus, development of sub-acute dementia, disturbances of alert, vision and memory. All patients showed hyperintensity in the basal ganglia and cortex in the magnetic resonance study, pseudo-periodic activity in EEG and elevated 14-3-3 protein in CSF. Additionally, CSF samples were studied from patients with other neurological symptoms, including dementia and tropical spastic paraparesis/HTLV-1 associated myelopathy (HAM/TSP). The dementia patients showed a chronic and progressive decline of intellect and behaviour, which caused a gradual restriction in their customary daily living activities. In addition, dementia patients showed cognitive impairment associated with systemic organic disease not related to Alzheimer diseases or CJD. The diagnosis of HAM/TSP was made according to the World Health Organization guidelines [45]. Other known causes (i.e., multiple sclerosis, spinal cord compression) of progressive spastic paraparesis were excluded. All HAM/TSP patients were positive for the HTLV-I viruses.

**Table 1:** General information for CSF analysis.

	Number cases	M/F	Median age ± SD	Median age ± SDMale	Median age ± SDFemale
sCJD	35	11/24	59.0±10.0	60.9±6.2	58.7±11.5
fCJD	11	7/4	57.2±8.4	54.1±9.1	62.5±3.7
Dementia	3	1/2	51.6±5.5	52	51.5±7.7
Paraparesis	3	1/2	60.0±12.5	47	66.5±7.7
Healthy controls	16	12/4	63.7±11.0	63.8±12.0	63.5±8.9

In routine practice, a CSF analysis is performed in all patients with the suspected diagnosis of CJD. All patients gave written informed consent to participate in the study as part of a secondary analysis after 14-3-3 levels were tested as a diagnostic practice. The study was conducted according to the provisions of the Helsinki Declaration, and was designed in accordance with the relevant Chilean legislation and was carried out with the approval of the Ethics Committee of the El Salvador Hospital, Santiago, Chile. All samples were manipulated in a level II biosecurity facility. Histological analysis of human post mortem samples was approved by the Ethics Committee of the Faculty of medicine of the University of Chile and FONDECYT funding agency (protocol number CBA #0323 FMUCH).

### CSF protein analysis

CSF samples were collected in polypropylene tubes, centrifuged at 2500 rpm for 5 min to eliminate possible contamination with cells or tissue, and the supernatant was stored at −80°C prior to analysis. For this study the presence of 14-3-3 protein, PrP and active LDH was analyzed. 14-3-3 and PrP protein levels in CSF were measured after analysis of 20 µl of sample by Western blot analysis using standard methods [46]. Dilutions of antibodies used: rabbit polyclonal anti-14-3-3, 1∶1000 (Santa Cruz Biotechnology, Cat.No. sc-629) or mouse monoclonal anti-PrP (3F4), 1∶3000 (COVANCE, Cat. No. SIG-39620); secondary antibodies were horseradish peroxidase-conjugated anti-rabbit (Invitrogen) or anti-mouse antibody (Zymed) at a dilution of 1∶3000. Positive and negative controls were included in each Western blot run. As a 14-3-3 positive control a HEK-293 cell lysate was used. As negative controls CSF from healthy individuals or patients with either a clinical or pathological diagnosis of an alternative disease were used. Membranes were developed by enhanced chemiluminescence assay (Amersham Biosciences, Cardiff, UK). Protein bands were quantified by densitometry on films with a non-saturated signal using ImageJ software. CSF LDH activity was measured using a commercially available kit (Biovision, Cat.No. K311), according to the manufacturer's recommendations in combination with a LDH standard (Cayman Chemicals, Cat.No. 10009321).

### Proteinase K (PK), Thermolysin, and PNGase F treatments

PK assays were performed using an adapted protocol we previously described [47], 20 µl of CSF samples were treated 30 min at 37°C with different concentrations of PK (0.5, 1 and 2 µg/ml). Proteolysis was stopped by adding phenylmethylsulfonyl fluoride followed by SDS-sample buffer and heating for 5 min at 95°C. In thermolysin assays, 20 µl of CSF samples were treated 30 min at 70°C with different concentrations of thermolysin from *Bacillus thermoproteolyticus rokko* (Sigma–Aldrich). Proteolysis was stopped by adding SDS-sample buffer and heating for 5 min at 95°C. For deglycosylation, 20 µl of CSF samples were treated with N-glycosidase F (PNGase F) (Biolabs) following manufacturer's recommendations. Briefly, after addition of denaturation buffer CSF samples were incubated for 5 min at 95°C. Samples were cooled to 25°C, and then the reaction buffer and 10 U of *N*-glycosidase F (PNGaseF) (BioLabs) was added. After 1 h at 37°C, sample buffer was added and samples were heated for 5 min at 95°C. Samples were analyzed by Western blot. As a positive control, a human post mortem sample derived from brain cortex of a healthy control individual was analyzed. Frozen brain tissue was obtained from the Harvard Brain Tissue Resource Center (Boston, USA http://www.brainbank.mclean.org/) and then processed for biochemical analysis by homogenization of equivalent amounts of tissue in PBS containing protease and phosphatase inhibitors with further dilution in RIPA buffer.

### Histology analysis

Ten micrometer-thick sections were obtained from formalin fixed, paraffin embedded blocks of the brains of CJD and control patients. The paraffin-embedded sections were deparaffinized in xylene, followed by rehydration in a decreasing concentration of ethanol solutions. For routine pathological examination, deparaffinized sections from all blocks were stained with hematoxylin and eosin stains and Luxol Fast Blue. Sections for immunohistochemistry were incubated in 10 mM citrate sodium buffer (pH 6.0) and heated in a microwave oven three times for 5 min for antigen recovery. Washed in TBS IHC wash buffer and treated with formic acid for 5 min and washed again. Sections were pretreated with 0.3% H_2_O_2_ in methanol for 30 min at room temperature to inhibit endogenous peroxidase activity. After washing two times with TBS IHC wash buffer for 5 min, sections were blocked with 0,3% normal horse serum for 30 min at room temperature, followed by incubation with anti-14-3-3 (1∶1000) and mouse monoclonal anti-PrP (3F4) (1∶1000) in a humidified chamber at 4°C overnight. Negative control sections were incubated with a negative control reagent (Dako) instead of primary antibodies. After washing two times with TBS IHC wash buffer for a total of 5 min, the sections were incubated with biotinylated secondary antibody for 30 min at room temperature, and rinsed two times with TBS IHC wash buffer for a total of 5 min, followed by incubation with an ABC kit (Vector) for 30 min at room temperature. After rinsing with, TBS IHC wash buffer, peroxidase labeling was visualized with DAB (Impact DAB, Vector) for 3 min at room temperature. Sections were rinsed in tap water for 10 min, dehydrated, cleared and mounted.

### Statistical analysis

Data was analyzed by parametric t-test (two-tailed) and significance was expressed as follow: * *p*<0.05; ** *p*<0.01; *** *p*<0.005; n.s.: non significant. For the statistical analyses SigmaPlot and GraphPad software were employed.
